# Cellular Senescence as the Pathogenic Hub of Diabetes-Related Wound Chronicity

**DOI:** 10.3389/fendo.2020.573032

**Published:** 2020-09-16

**Authors:** Jorge A. Berlanga-Acosta, Gerardo E. Guillén-Nieto, Nadia Rodríguez-Rodríguez, Yssel Mendoza-Mari, Maria Luisa Bringas-Vega, Jorge O. Berlanga-Saez, Diana García del Barco Herrera, Indira Martinez-Jimenez, Sandra Hernandez-Gutierrez, Pedro A. Valdés-Sosa

**Affiliations:** ^1^The Clinical Hospital Chengdu Brain Sciences Institute, University of Electronic Science and Technology of China, Chengdu, China; ^2^Tissue Repair, Wound Healing and Cytoprotection Research Group, Biomedical Research Direction, Center for Genetic Engineering and Biotechnology, Playa, Cuba; ^3^Cuban Neurosciences Center, Playa, Cuba; ^4^Applied Mathematics Department, Institute of Mathematics, Federal University of Rio de Janeiro, Rio de Janeiro, Brazil; ^5^Department of Surgery, Hospital Salvador Allende, Havana, Cuba

**Keywords:** senescence, proliferative senescence, chronic wounds, diabetic ulcers, aging

## Abstract

Diabetes is constantly increasing at a rate that outpaces genetic variation and approaches to pandemic magnitude. Skin cells physiology and the cutaneous healing response are progressively undermined in diabetes which predisposes to lower limb ulceration, recidivism, and subsequent lower extremities amputation as a frightened complication. The molecular operators whereby diabetes reduces tissues resilience and hampers the repair mechanisms remain elusive. We have accrued the notion that diabetic environment embraces preconditioning factors that definitively propel premature cellular senescence, and that ulcer cells senescence impair the healing response. Hyperglycemia/oxidative stress/mitochondrial and DNA damage may act as major drivers sculpturing the senescent phenotype. We review here historical and recent evidences that substantiate the hypothesis that diabetic foot ulcers healing trajectory, is definitively impinged by a self-expanding and self-perpetuative senescent cells society that drives wound chronicity. This society may be fostered by a diabetic archetypal secretome that induces replicative senescence in dermal fibroblasts, endothelial cells, and keratinocytes. Mesenchymal stem cells are also susceptible to major diabetic senescence drivers, which accounts for the inability of these cells to appropriately assist in diabetics wound healing. Thus, the use of autologous stem cells has not translated in significant clinical outcomes. Novel and multifaceted therapeutic approaches are required to pharmacologically mitigate the diabetic cellular senescence operators and reduce the secondary multi-organs complications. The senescent cells society and its adjunctive secretome could be an ideal local target to manipulate diabetic ulcers and prevent wound chronification and acute recidivism. This futuristic goal demands harnessing the diabetic wound chronicity epigenomic signature.

## Introduction

Diabetes mellitus (DM) is a heterogeneous metabolic disease characterized by chronic hyperglycemia resulting from defects in insulin secretion, insulin action, or both (1). Despite the genetic underpinnings of the diseases, the prevalence of both type 1 and type 2 diabetes (T1DM and T2DM) is globally increasing at a rate that outpaces genetic variation and approaches to pandemic magnitude (2, 3). Like other age-related chronic diseases, diabetes is essentially impinged by the convergence of basic aging mechanisms that underlie age-related tissue dysfunction (4, 5).

Although more than 300 theories have emerged over the years to explain the intrinsic molecular and evolutionary drivers behind organismal aging (6); the onset of cellular senescence seems to act as a foundational pillar for multi-organs and organismal aging (6–9).

An intense debate has existed so far addressing whether senescence precedes or follows the onset of perpetual inflammation and insulin resistance (IR) (10). Irrespective to “who-precedes-who,” diabetic patients experience an obvious accelerated aging process that increases their susceptibility to morbidity and earlier mortality (10). Hence, diabetes-affected patients have a significantly shorter life expectancy than non-diabetic individuals (11, 12), while this life expectancy reduction is largely dependent on diabetes duration (2, 13).

Aside from the reduced number of fatalities caused by acute diabetic complications, the major clinical challenge of diabetes is the progressive and expansive morbidity and mortality resulting from the long-term secondary complications (14–16). Within the constellation of diabetic complications, the delayed and poorness in triggering and progressing along a physiological repair response is of major clinical significance (17, 18). As in most chronic wounds, the diabetic wound is frequently distinguished by its chronicity and by the asynchrony of the healing phases within a specific wound niche (17, 19, 20).

Diabetes undermines skin cells physiology and progressively intoxicates the dermal layer by the accumulation of advanced glycation end products (AGEs) and free radicals derivatives (21–23). Accordingly, most if not all of the events encompassed within the cutaneous healing process including hemostasis, inflammation, matrix deposition, angiogenesis, contraction, remodeling, and re-epithelialization are somewhat buffeted by diabetes (20, 24). Aside from the impaired healing response, a parallel dark arista of diabetic ulcer pathology is the high rate of ulcer recidivism after the primary wound closure (25, 26). In line with this fact, a classic study reveals that roughly 40% of patients have a recurrence within 1 year after ulcer healing. Thus, the epithelized ulcers are considered as being in remission rather than being healed (10). Additionally, an underappreciated risk of diabetic foot ulcers (DFU) recidivism is its ability to recur or “metastize” at anatomical locations distinct from the primary occurrence, often involving the contralateral limb which entails the risk of subsequent amputation within 5 years following a primary amputation (25, 27).

The synergy between compromised healing machinery and the predisposition to peripheral tissues infection nurture the alarming figures of lower extremity amputations (28). Thus, DFU has historically remained as the worldwide leading factor for non-traumatic lower extremities amputations (29, 30). These evidences support the David's Armstrong “cancer analogy” concept. The concept itself highlights that the 5 year mortality rates associated to foot ulceration and lower extremity amputation, surpasses those registered for five common cancers (31). Subsequent studies have further enriched the notion that lower extremity amputation reduces life expectancy in diabetics (32); and that ulcer severity is a more significant predictor of subsequent mortality than coronary and peripheral arterial disease, or stroke (33).

While healing diabetic wounds is instrumental for amputation prevention, there exists the underlying need to identify the molecular factors driving the secondary diabetic complication of impaired healing (20, 34–36). Hyperglycemia is the major systemic risk factor for the onset and progression of diabetic complications (37, 38); and mechanistically speaking, it is endowed with a proximal position in the diabetic biochemical environment where numerous endogenous stressors drive cellular senescence (7). Chronic low-grade inflammation and an increased burden of senescent cells are hallmarks of aging in diabetic subjects (10). Hyperglycemia *per se* is known to act as a senescence-promoting factor for cultured cells (39, 40), and steadily precipitates organs complications and functional demise by different mechanistic pathways (10, 41–44).

The notion that cellular senescence is an imperceptible underlying force in the pathogenesis of wound chronicity and ulcers recurrence has been accrued for years (45–48). Consequently, we suggest that diabetes-associated wound healing failure and reduced tissue resilience are clinical translations of an “entrenched” wound senescent cells society, with self-perpetuating and propagating abilities. Subsequent to the enlightening opinion article by Sahin and DePinho (49), which postulates a rational model depicting how mitochondria is intersected by different pro-senescence pathways—We decided to review the current knowledge on cellular senescence as a putative founding pillar for diabetic wound healing impairment. The search strategy involved Medline/Pubmed and other reference data sources as Google Scholar, Scielo, Bioline International (www.bioline.org.br), etc; introducing key words as: chronic wounds+senescent cells, diabetes+senescence, DFU, mitochondria, telomeres erosion, replicative senescence+chronic wounds, fibroblasts+proliferative arrest. In first instance, we delineated the major molecular factors invoked as diabetes-associated senescence drivers in a systemic level and attempted to extrapolate the impact of these hallmarks for the wound microenvironment. The contemporary evidences describing the participation of granulation tissue senescent cells and the impact of diabetic-senescence drivers in mesenchymal stem cells biology were reviewed. All the articles reviewed were limited to English language and with no date restriction. Thus, classic articles dated on the 70's and 80's are referenced.

## Diabetes and Cellular Senescence. Brief Mechanistic Overview

Conceptually, aging is defined as an evolutionary process with a time-dependent functional decline that affects all higher organisms. Since cellular senescence contributes to organismal demise aging is associated to morbidity and mortality. Aging therefore involves different interdependent hallmarks on cellular, molecular, and organism levels (50, 51). Senescence is a cellular program that induces proliferative arrest accompanied by morphological modifications, metabolic reprogramming, implementation of a complex proinflammatory spreadable secretome, increased autophagy, apoptosis resistance, and epigenetic reprograming (52–54).

Diabetic environment is overwhelmed of multiple cellular senescence-contributing factors that ultimately translate in organismal aging with well-known nosogenic consequences (7, 41, 55). Diabetes is therefore representative of the model that “organisms accumulate modified macromolecules” and that these macromolecules increase over time and interacts with proteins and tissues, inducing structural changes generating the so called “damage accumulation” (56, 57). As elegantly described by Palmer et al., the fundamental aging mechanisms broadly fall into the following categories: (1) macromolecular dysfunction, (2) sterile inflammation, (3) progenitor cell dysfunction, and (4) cellular senescence (58). Of note, these four mechanisms are represented in diabetes and reciprocally link aging and diabetes (58, 59). Accordingly, many aging hallmarks appear earlier or are overexpressed in T2DM, including the “inflammaging” as other forms of cellular stress: oxidative stress, mitochondrial dysfunction, and endoplasmic reticulum (ER) stress (10, 58, 60).

**Figure 1:**
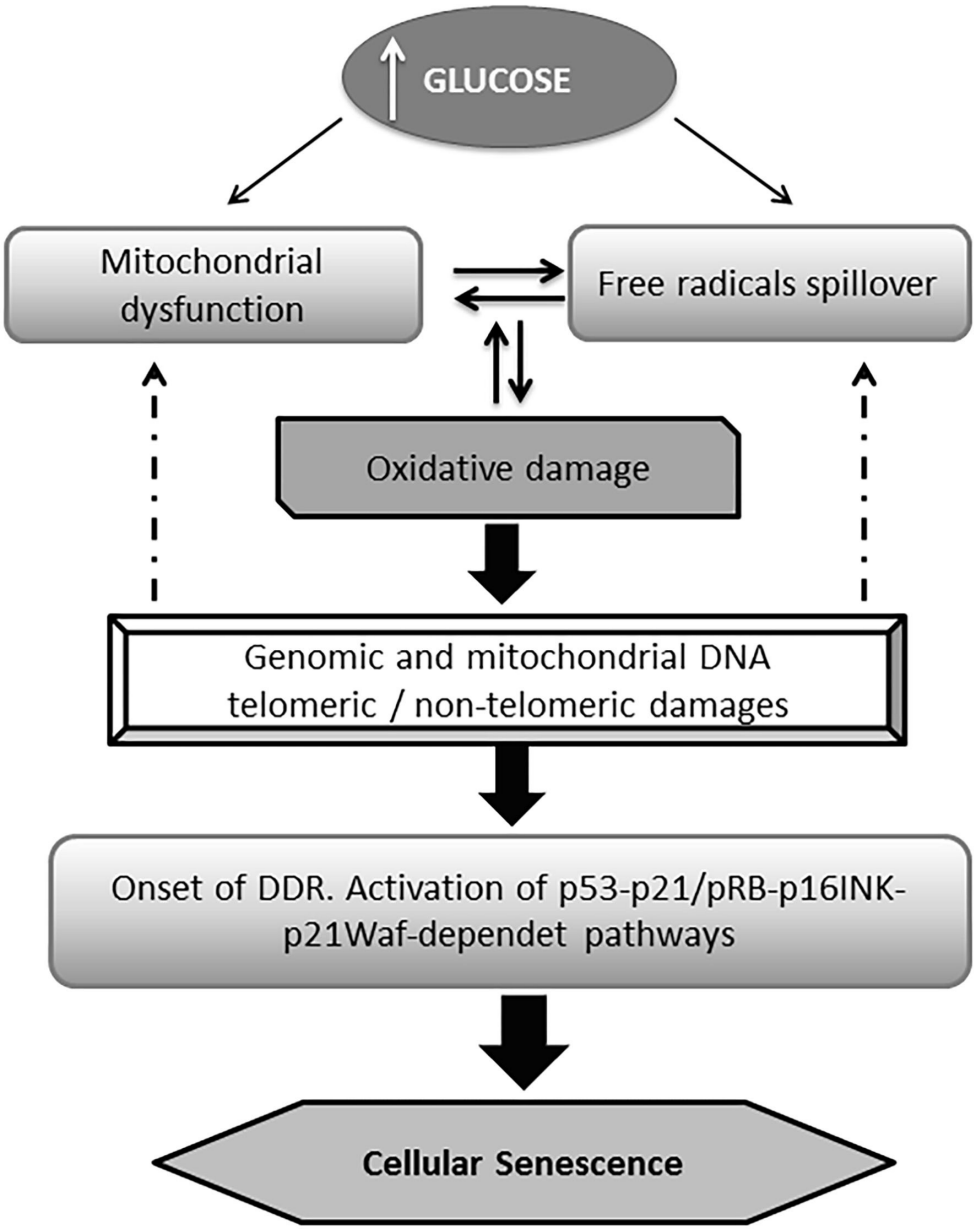
General pathway of glucose-induced senescence. Multiple evidences converge to indicate that high glucose drives premature senescence acting as a proximal trigger for a myriad of subsequent biochemical derangements. Hyperglycemia is ensued by an excessive mitochondrial production of reactive oxygen species (ROS) which in turn generates the typical pro-oxidative environment of diabetes. Alike, mitochondrial dysfunction derives from the ROS-mediated attack to its DNA, and the structural damage to respiratory chain proteins which generates a destructive vicious circuitry. Senescent burden can be multiplied in neighboring cells via the local oxidative milieu. The DNA chemical damage (telomeric and non-telomeric) induced by the impact of ROS is followed by the activation of the DNA damage responses (DDR) pathway; which in turn activates the p53 and/or the p16INK4A pathways. The later facilitates the accumulation of phosphorylated pRb eventually accounting for cell senescence program orchestration.

Hyperglycemia which also comprises the oscillation of the “trivial” blood glucose levels, known as hyperglycemia transient spikes, is a major risk factor for a variety of pathologies and clinical and surgical complications (61, 62), including poor wound healing and wound infections (63–65). Hyperglycemia is also the proximal trigger for all the downstream molecular derangements in diabetes including cellular senescence (66, 67). The excessive generation of ROS and the expansive oxidative attack to multicellular targets including mitochondrial structures, generate an interdependent and perpetuative loop. Mitochondrial and genomic DNA damages including or not telomeric regions trigger different effector pathways that provoke cell senescence and proliferative arrest (Figure 1) (10, 68–72).

**Figure 2:**
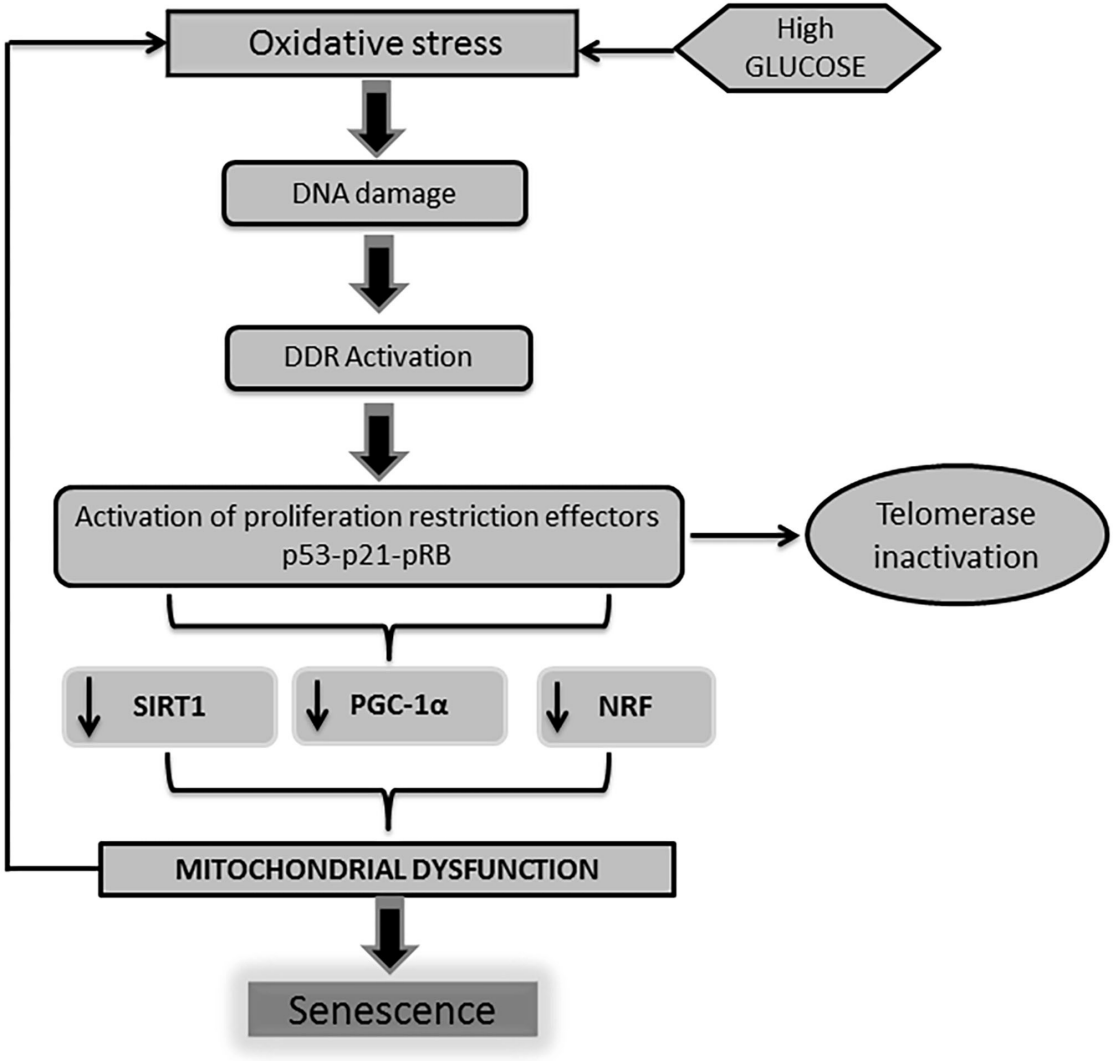
DNA damage is linked to mitochondrial physiology. As described earlier, high glucose levels trigger a cascade of events leading to the activation of professional inhibitors of cell cycle proliferation as p53-p21-pRB. The activation of p53 by DNA damage response (DDR) impacts on mitochondrial physiology and energy metabolism. Telomeric shortening negatively influences mitochondrial homeostasis. This mitochondrial failure mostly derives from the p53-mediated repression of peroxisome proliferator-activated receptor gamma co-activator 1α/β gene (PGC-1α/β) expression. Of note, PGC-1 is largely involved in mitochondrial biogenesis and function, including oxidative phosphorylation and reactive oxygen species (ROS) detoxification. Along with PGC-1 activity abrogation, other mitochondrial biogenesis regulators as nuclear respiratory factor (NRF) and SIRT-1 are also impaired. Here again, pathogenic vicious circles are established leading and amplification of cells senescence as a final outcome.

From the molecular point of view, two major basic operators have been described for the onset of the cellular senescence phenotype derived from the impact of a high glucose burden: (1) p53-p21 and (2) pRb-p16INK4a-p21Waf1 (73–75). Moreover, convergent lines of evidence point to the role played by p53 in the regulation of cell cycle arrest and senescence (50, 76, 77). It has been recently shown that telomeric damage activates a p53-dependent program which leads to mitochondrial failure via the repression of peroxisome proliferator-activated receptor gamma co-activator 1α (PGC-1α) gene expression (78). PGC-1α is a master regulator of mitochondrial biogenesis and function, including oxidative phosphorylation and (ROS) detoxification (79). Other cognate proteins involved in mitochondrial biogenesis and function are also impaired upon the orchestration of the DDR, which ultimately amplifies mitochondrial functional impairment (Figure 2).

Likewise, the retinoblastoma protein (pRb) plays a central role toward the onset of senescence. The cell cycle inhibitors p16INK4a and p21Waf1 cooperate to keep pRB in its active hypophosphorylated/growth inhibitory state (80, 81). Of note, nuclear overexpression of p53 phosphorylated on serine-15 and p21Cip have been detected in fibroblasts derived from reluctant-to-heal DFU, supporting the notion that granulation tissue fibroblasts-proliferative arrest, promote cellular senescence and fibrovascular stagnancy (82). Similarly, cultured diabetic cutaneous fibroblasts also exhibit an exaggerated activation of the p53/p21-dependent pathways along with a significant increase in senescence-associated β-galactosidase (SA-β-Gal) activity and phospho-γ-histone H2AX (pH2AX) level, as indicative of cellular senescence and impaired wound healing (83). Outspoken morphological and biochemical alterations in diabetic ulcer and diabetic intact skin-derived fibroblasts are described, which are all suggestive of impaired proliferative capability (84–86). A seminal study by Vande Berg et al. confirmed the existence of senescent fibroblasts in pressure ulcers, which exhibit limited proliferative capability (87). Finally, senescent cells are readily distinguished in addition to their blunted proliferative activity (88, 89), by their larger size and flattened morphology, and an altered gene expression including upregulation of SA-β-Gal and pro-inflammatory chemokines and cytokines (90, 91).

## The Senescence Messenger Organ, Metabolic Derangements, and Oxidative Stress on Wound Cells Biology

**Figure 3:**
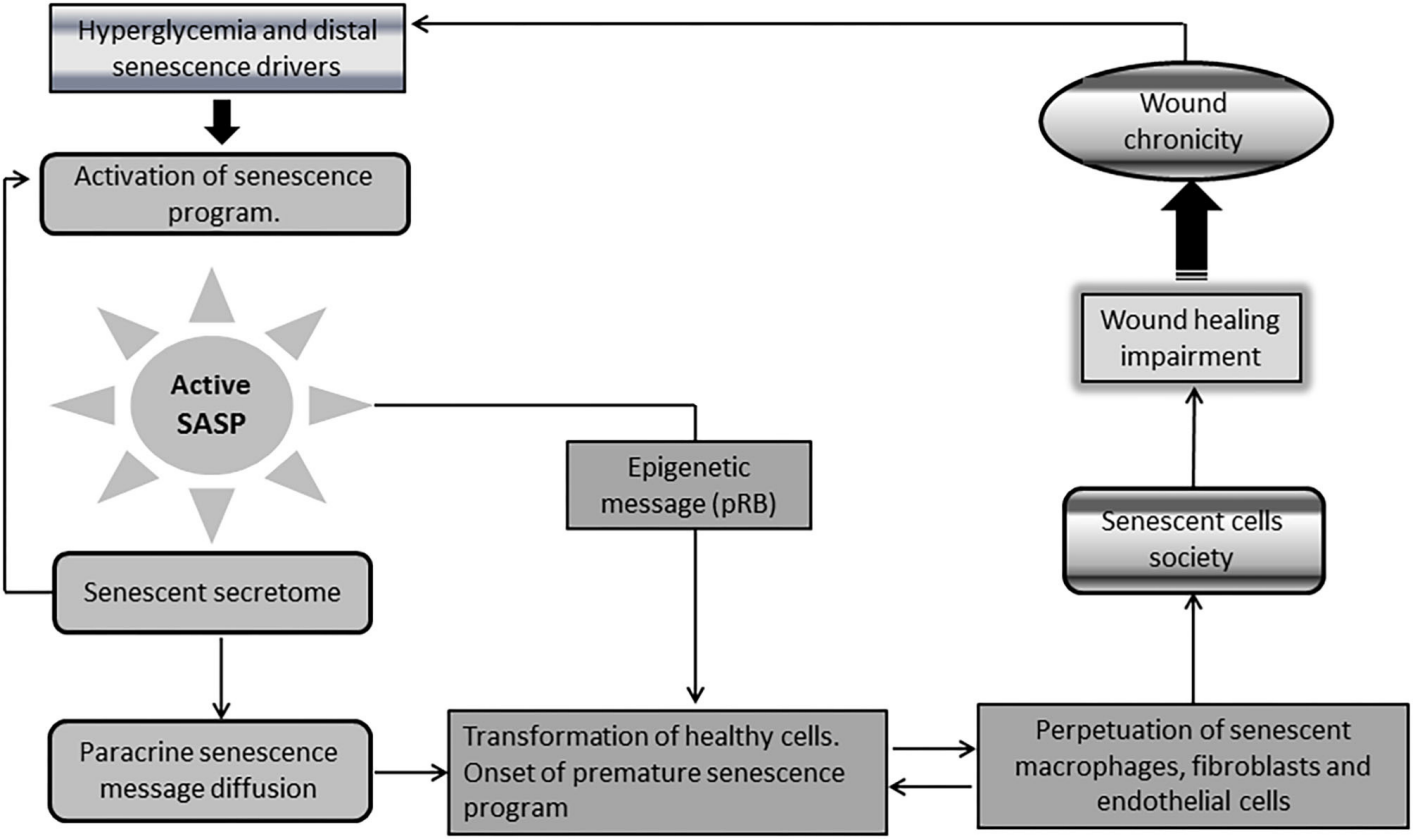
Chronic wounds and the society of senescent cells. A singular and distinctive marker for senescence cells is the onset of a pro-inflammatory program identified as senescence-associated secretory phenotype (SASP). Diabetic senescent cells SASP are typically endowed with inflammatory effectors and matrix degradative enzymes. The paracrine release of this secretome accounts for the diffusion of a progeroid message and ultimately engenders a threshold of senescent cells within the wound via transformation of normal neighbor cells into senescent cells. SASP also spread the epigenetic signature ingredients of senescence which further contributes to expand and perpetuate the so called “senescence cells society” Accordingly, irreversibility of senescence is largely dependent upon pRB epigenetic events. It is therefore plausible that epigenetic mechanisms contribute to shape a long-lasting epigenetic memory for SASP, and the perdurability of the senescent society. The society integrates within the wound bed to M1-polarized macrophages, as arrested fibroblasts, and endothelial cells that “contaminate” keratinocytes leading edge, thus impairing wound closure and promoting wound chronicity. Given that the diabetic chronic wound is constant source of inflammatory mediators and ROS, insulin resistance is amplified leading to more hyperglycemia. This is a major perpetuative pathogenic circuitry. These factors nurture the existence and perdurability of the.

A singular and distinctive marker for senescent cells is the onset of a pro-inflammatory program identified as *senescence-associated secretory phenotype* (SASP) (92, 93). This “messenger organ” is able to reinforce the cellular proliferative arrest, and spread different types of senescence messages integrated in the senescence-messaging secretome (SMS) (94). SASP ingredients are mostly constituted by interleukins, chemokines, growth factors, secreted proteases, and secreted insoluble proteins/extracellular matrix (ECM) components (92, 95, 96). Hyperglycemia-induced senescence is associated to a typical diabetic SASP. Reciprocally, SASP further enhances insulin resistance (97), amplifies diabetes-related endovascular and tissue inflammation, and ultimately disseminate a senescence message that impairs diabetic wound matrix accumulation (44). This archetypical SASP transforms healthy cells into aged cells, and perpetuates the residence and the turnover of the senescence cells society and of inflammation- polarized macrophages (44, 98–102) (Figure 3).

By the contrary, SASP attenuation prolongs health span and lifespan of diabetic mice and slows diabetes progression and/or the development of its different complications (44). Of relevance for the tissue repair process, SASP cocktail contains growth factors and extracellular matrix-remodeling proteases with important functions across the different overlapping phases of the healing process (103). Via the paracrine activity of its soluble senescence messages, SASP may disrupt tissues homeostasis provoking a variety of age-related pathologies and disabilities (95, 104). Surprisingly, two well-reputed growth factors implicated in wound fibro-angiogenic response: transforming growth factor β1 (TGF-β1) and vascular endothelial growth factor (VEGF), are involved in the remote transformation of healthy to senescent cells (105, 106).

Although there is relative scarcity of information regarding the identification or quantification of senescent cells-extracellular vesicles cargo (95); important findings have revealed the link between extracellular miRNAs and the development and progression of T2DM complications. About 40 circulating miRNAs are significantly deregulated and implicated in diabetic endothelial dysfunction, inflammation, cellular senescence (107), and in the pathogenesis of diabetic impaired wound healing (108, 109). Diabetic wounds are typically associated with a persistent inflammatory infiltration that involves accumulation of senescent macrophages. These cells are key effectors for the clearance of senescent cells, and their absence or dormancy may favor a SASP with sustained inflammatory message (48). In line with this, it has been observed that aged and diabetic wounds populated with senescent macrophages produce a CXCR2-enriched SASP. Importantly, CXCR2 is an inflammatory chemokine that drives primary human dermal fibroblasts toward a pro-fibrotic and senescent phenotype, via the paracrine induction of nuclear p21 (53). Furthermore, clinical samples of diabetic wounds exhibit a protracted population of M1 phenotype polarized senescent macrophages, driven by the expressions of NLRP3, caspase1, and IL-1 (110). Taken together these evidences incite to reinforce that: (a) both diabetic and aged wounds are chronically infiltrated by inflammatory mononuclear cells, (b) that diabetes seems to disrupt the physiological fine tuning for macrophages turnover and polarization, (c) these population of inflammation-polarized macrophages (M1) contribute to impaired healing, become senescent, and impose senescence to other granulation tissue cells via an inflammation loaded-SASP (111–114). Once again, the pathogenic synergistic link between chronic inflammation and cellular senescence, toward the implementation of a pathological wound repair is further strengthened.

On the opposite extreme, an elegant study by Birgit Ritschka et al. uncovered the benefits of a transient exposure to SASP by inducing cell plasticity, stemness, and regeneration. Thus, it was anticipated that transient induction of a senescent phenotype produces a “wound healing-promoting SASP” (92). For the scenario of an acute cutaneous healing in which ordinarily senescent fibroblasts and endothelial cells appear few days after skin injury, the SASP-derived platelet growth factor (PDGF)-AA stimulates wound closure via myofibroblast differentiation. Accordingly, there are circumstances in which SASP may exercise a positive role in normal wound repair (115). Likewise, the acute exposure of human dermal fibroblast to hydrogen peroxide successfully induced fibroblasts senescence and an archetypal SASP, which stimulated keratinocytes migration and myofibroblast transition via increased secretion of growth factors including different isoforms of PDGF, TGF-β, and VEGF. Again, this finding indicates that a population of senescent fibroblast from acute wounds, may positively modulate the wound healing process (116).

Chronic diabetic wounds show marked differences not only compared to acute non-diabetic wounds, but also between both chronic healing, and non-healing diabetic wounds (117–119). Given that senescent cells may have diverse and context-dependent effects on tissue, the existence of two different types of SASP is predictable. One associated to acutely induced and physiological healing wounds, and the concomitant to chronic wounds—as diabetic foot ulcers in which the secretome is basically inflammatory and anti-proliferative. Therefore, SASP is a dynamic and malleable organ whose phenotype appears to act in a tissue-specific manner, within a temporary window, and vectoring particular chemical and epigenetic signatures (120–122).

In the realm of cellular metabolism, it is known that metabolic dysfunction is a powerful organismal driving force for aging, and a senescence hallmark (123) with meaningful repercussion in diabetes (59, 124). Diabetes *per se* is a metabolic disease in its basic origin, but that progress along with a continuous deterioration of proximal metabolic regulators such as PGC-1α, SIRT 1, and mTOR (78, 79, 125–132). PGC-1 plays an essential role as a master metabolic regulator as mitochondrial biogenesis and function, oxidative phosphorylation, and ROS detoxification (79). PGC-1α axis failure contributes to glucose intolerance, mitochondrial bankruptcy, systemic chronic inflammation, amplified pro-oxidative environment, and ultimately to organismal aging (133, 134). SIRT1 is a prominent member of a family of NAD-dependent enzymes that inhibits transcriptional factors, such as p300, NF-κB, P38MAPK, Histone 3, MMP-9, FOXO3a, and most importantly to p53 (135). SIRT1 exhibit numerous and diverse biological functions including the regulation of energy homeostasis, cell cycle, inflammation, oxidative stress threshold, ER stress-related apoptosis, and ultimately a senescence-preventive factor (136, 137). Conclusively, both PGC-1 and SIRT1 alone or in physiological synergy are key modulators of molecules and pathways involved in cellular senescence. This anti-senescence guardian role is played by different interconnected mechanisms, that protect and generates mitochondria, reduce tissue inflammation, and combat oxidative and nitrosilative stress (138–142).

Recent evidences document that SIRT1 activation accelerates and improves the wound healing response in diabetic mice by promoting angiogenesis, via an angio-protective effect against oxidative stress injury (143). In line with this, the SIRT 1 agonist Resveratrol has shown to reduce the hyperglycemia-triggered endothelial dysfunction, to stimulate angiogenesis and consequently wound closure in db/db mice. Again SIRT 1 axis activation reduces oxidative stress and favors downstream pro-angiogenic pathways (144). Furthermore, two anti-aging proxies like metformin and resveratrol have proved to stimulate wound healing in aged animals, by preventing age-related AMPK deactivation, and attenuating age-associated angiogenic inhibition (145). These experiments confirm the pro-senescent driving effect of ROS-oxidative stress and their negative impact for wound healing biology, especially in diabetic organisms (143, 146–149).

Mitochondria, routinely identified as the powerhouse of the cell are the major generator of members of the ROS family such as superoxide anion, hydroxyl and peroxyl radicals, and hydrogen peroxide (150–153). Furthermore, ROS/oxidative stress/mitochondrial dysfunction and inflammation behave as an indissoluble, interconnected nosogenic unit that links organismal aging, T2DM, and poor wound healing (73, 154–158). Converging evidences demonstrate that high levels of ROS disrupt the normal healing process (159–161) and constitute a hallmark in chronic wounds including recalcitrant DFU (147, 159, 162). In this scenario ROS overproduction is associated to the upregulation of cell cycle inhibitors as to positivity for the SA-β gal marker. The two most studied cell cycle proliferation inhibitors the p53-p21 and the p16-Rb pathways are responsive to ROS-induced telomere erosion, and to non-telomeric DNA damage (SIPS) via oxidative stress (7, 163). Accordingly, oxidative stress-influenced events translate in a collection of wound cells senescence attributes as proliferative arrest, fibroblasts reduced migration, imperfect and poor angiogenesis, amplified apoptosis of granulation tissue productive cells, extended inflammatory polarization, and ultimately wound chronicity (164–166).

Glycolysis is enhanced as cells undergo replicative senescence. Glycolytic enzymes are a major target of oxidation and modification by AGEs, and ROS products during replicative senescence (167). This accounts for a fall of ATP and GTP intracellular levels (167). Mechanistically, enhanced glycolysis contributes to the development of a senescent phenotype (168, 169); as it is the case for the diabetic abnormal increase in glycolysis which seems to drive vascular aging (170). Experiments from two decades ago illustrated that normal cutaneous fibroblasts became arrested and reluctant to proliferate, when they were exposed T2DM-wound fibroblasts conditioned media. This proliferative arrest was associated to decreased DNA content, and was proportional to L-lactate production and incorporation of D-glucose in the media (171, 172).

Conclusively, diabetic metabolic and biochemical derangements as glycolysis are drivers for the onset of cellular senescence and organismal aging. Metabolic dysfunctions trigger a wave of molecular derangements where glucooxidative and nitrosilative stress, inflammatory, and cellular senescence, intersect and cooperate for the implementation of a wound chronicity profile.

## Cellular Senescence in Diabetic Sweet Environment

The role of cellular senescence within the complex pathogenic realm of T2DM dates back to the 70's and 80's of last century. The inspiring theory stated the existence of a kind of a pre-senescent state in fibroblast from pre-diabetic subjects which appear to predetermine the wound healing failure (45). This “pre-senescent state” notion has remained effective and validated nowadays (173), and has been expanded to extracutaneous cells (60). Aside from the impact generated by the exposure to glucose spikes which enforce pro-senescent and apoptogenic signatures in most skin cells (45), and even cerebral cells populations (174); these germane studies allow to infer that a persistent heritable abnormality, related to a precocious cellular senescence is present in mesenchymal tissues of genetically predisposed subjects to suffer of diabetes (45). The forces that “erase” from the core of the cell the intrinsic mechanisms to migrate and proliferate within a physiological repair response remain unclear (175–177). Nevertheless, local fibroblasts senescence has been invoked as a major wound healing deterrent factor (53, 178, 179); particularly when a senescent cells threshold is reached per tissue area (180, 181).

The skin is the preferential target organ for extrinsic and intrinsic aging driving forces which account for keratinocytes and fibroblasts to precocious decline (182). Skin fibroblast is a mesenchymal-derived cell with a pivotal role in wound repair. It is difficult to conceive granulation tissue creation, contraction, and basement membrane elaboration in a mammal devoid of this type of cells (183, 184). Cutaneous fibroblasts are sensitive cells, impacted by a variety of stressors which may modify their biological behavior and ultimately the wound healing course (185, 186). High glucose levels and their adjacent biochemical derangements act as a major skin cells pro-senescent factor (7, 187, 188). Even in non-diabetic subjects, high glucose levels are associated to an apparent cutaneous advanced age (189). Proliferative refractoriness, torpid migration capabilities, susceptibility to apoptosis, and limited secretion of extracellular matrix constituents, are hallmarks of diabetics' cutaneous fibroblasts (165, 190). These hallmarks have been historically reproduced *in vitro* when healthy donor-derived cells, are exposed to high glucose burden stress (63, 171, 191, 192). These fibroblasts adverse behavior is over-represented in DFU, and represent active pieces within the pathogenic puzzle of wound chronicity (17, 193, 194). Classic studies by Miriam Loots and co-workers illustrated that fibroblasts isolated from the ulcer bed of T2DM-donor patients, exhibit a diminished proliferative capacity and an abnormal morphology (84). This observation became paradigmatic when converging studies reproducibly showed that human cutaneous, renal, and periodontal fibroblasts cultured under “hyperglycemic” conditions, expressed a variety of premature cellular senescence markers including inhibition of spontaneous and growth factors-stimulated proliferation (20, 195–198).

Influential studies of late 70's indicated that high glucose concentrations directly or indirectly damage cell's DNA, induce telomeric attrition and other forms of DNA damage in cultured cells (199), These findings have been recently substantiated including the toxic effects of AGEs (200, 201). The early *in vitro* models founded the classic thesis of a “point-of-no-return,” beyond which hyperglycemia resulted in irreversible progression to premature senescence (202). Form those years it was also known that cutaneous fibroblasts derived from insulin-dependent or insulin-independent diabetic patients, exhibit abnormal replicative capacity *in vitro* and that senescence, was far more precocious than in non-diabetic control subjects (203). Another sequence of enlightening *in vitro* experiments documented that diabetic' cutaneous fibroblasts exhibited impoverished synthetic and secreting capabilities (192, 204). By the contrary, glucose restriction to cultured human diploid fibroblasts inhibited the onset of premature senescence, and extended mean survival days and lifespan (205). It was later shown that when normal subjects-derived fibroblasts were challenged with the conditioned media from T2DM counterparts, proliferation was inhibited in a dose-dependent manner. Accordingly, this proliferative arrest turned a “reproducible and transmissible trait” which nurtured our hypothesis on the existence of a “senescence memory-transmissible factor” (88).

Premature cellular senescence has also been observed in endothelial cells exposed to a “diabetic environment” recreated by exposure to glycated collagen I. Although these cells did not show telomeric dysfunction, the process appeared presided by an increased expression of p14 and p53 consequent to an excessive oxidative stress (39, 206). Similarly, adipocytes subjected to glucose oscillations elicit an oxidative environment that damaged telomeres, and increased the presence of p53, p21, as pro-inflammatory cytokines (207). High glucose also impairs cell migration. Fibroblasts from diabetic mice migrate 75% less than those from normoglycemic controls, and display a defective response to hypoxia, a condition commonly present in chronic wounds (208, 209). Furthermore, skin keratinocytes exposed to high glucose also exhibit abnormal cellular morphology, insulin resistance, and decreased proliferation (66, 210). Taken together these experimental data support the clinical statement that abnormal levels of glycated hemoglobin and fasting glucose, are associated to poor ulcers healing and higher figures of lower extremity amputation in diabetics (211). Conclusively, fibroblast, endothelial cells, and keratinocytes –the most important cells for skin injury repair– are sensitive to hyperglycemia and oxidative stress, which act as the main steering factors toward senescence (202, 212).

## Wound Cells-Senescence Induction and Maintenance

Acute senescence seems to be a programmed process that is triggered in response to discrete stressors. It is established with fast kinetics and normally contributes to tissue homeostasis and repair (115). As described earlier, this type of acute injury-induced senescent response is endowed with its own SASP/secretome with wound healing promoting activity (115, 213). In contrast, chronic senescence may result from long-term unscheduled damage, and it is often associated with detrimental processes such as the creation of a stable society of senescent cells (90, 214).

Senescence maintenance poises as a relevant and futuristic target given its clinical implications for novel diagnostic procedures and therapeutic approaches. As most biological process, the acquisition of a senescent phenotype may be a progressively escalating, multistep event (215), what suggests that the molecular operators eliciting senescence are not necessarily those involved in its perpetuation. Accordingly, the chronic senescent phenotype is dictated by epigenetic mechanisms that ensure its long survival within the wound bed. The existence of a clinical correlation between a quantitative *in vitro* senescent cells population and a time-to-healing, support the notion of a perpetuated senescence society (216, 217). Mathematical simulation models have *in silico* predicted the dynamics of a senescent cells society. It is concluded that within a week time period a cell has an 83.22% possibility of entering a pre-senescent state if senescence soluble instructions are received from a neighbor senescent cell (218). This suggests the existence of a continuous population turnover with a stable senescent phenotype.

Diabetic ulcer cells are chronically exposed to major stressors that sculpt wound cells phenotype. In this respect, the local impact of a metabolically active SASP is likely to be pathogenically crucial via its SMS. A living, active SASP secretome would guarantee a constant pro-senescent cells threshold toward the establishment of a chronicity phenotype. In support to this observation is the fact that cells isolated from chronic wounds, become incompetent to upregulate those chemokines and cytokines involved in chronic inflammation resolution (219). Of note, cultured senescent fibroblasts in addition to be proliferative incompetent, decrease the synthesis and drive the degradation of matrix components through the expression of SASP ingredients (196, 220). This behavior suggests the persistence and dominance of the senescent imprint even after separated from the donor organism. These elements together again emphasize on the hypothesis of an existing “senescence phenotype memory.” This alleged memory may justify why wound bed cells subjected to “ideal” tissue culture conditions recreate the same behavioral traits as when in the donor's organism (165). In line with this we and other groups, have accumulated evidences supporting that wound senescent cells are able to reproduce and transmit their original morphological and functional phenotypic traits to their progeny.

1. Young granulation tissue of neuropathic diabetic ulcers reproduces a collection of senescent vascular defects observed in distant, intact, dermal vessel that take years-long for evolvement. The driving forces behind the “inheritance” of these progeroid morphological traits are likely associated to an epigenetic senescence signature (221).
2. Similarly, diabetic granulation tissue although being a “*de novo*” and short-lived “welding tissue,” its cells exhibit a sort of genetic or epigenetic imprinting for the deranged expression of glucose metabolism-related genes, which are implicated in insulin resistance, cellular senescence, and T2DM pathophysiology (221).
3. The *in vivo* proliferative arrest shown by ischemic ulcers fibroblasts is maintained even when cultured under “ideal physiological” conditions. This *in vitro* proliferative arrest coincides with the transcriptional upregulation of p53 mRNA, and its nuclear translocation with phosphorylation on serine 15. Ultimately these cells exhibited a conspicuous expression of p21Cip along with a marked under-expression of cyclin D1. Taken together, these findings describe molecular ingredients of senescence (82).
4. Classic studies by other groups also document the involvement of senescent phenotype fibroblasts derived from pressure ulcers in the mechanism of wound chronification. Accordingly, although these cells are cultured under standard conditions and remain viable, still they exhibit proliferative arrest as *in vivo*, being unable to complete DNA synthesis (87). Remarkably, elimination of pressure/ischemia stressors does not guarantee the resumption of a physiological healing trajectory (178).
5. Paradigmatic evidence on the existence of a senescent perpetuation program within the ulcer is the need for frequent sharp debridement (178). This procedure is ultimately addressed to remove proliferative senescent cells which hamper wound closure (178, 222). In this scenario Stojadinovic and co-workers demonstrated that c-myc overexpression is implicated in keratinocytes proliferative and migration arrest (223).

In conclusion, senescent phenotypic traits are transmitted and reproduced to descendent cells within the diabetic ulcer bed, and do not seem to fade away along successive generations' of *in vitro* passages.

## Diabetics' Stem Cells are not Exempted From Senescence

Mesenchymal stem cells (MSCs) are multipotent cells with a fibroblast-like morphology that are considered a lifelong cellular reservoir to ensure the continuous generation, replacement, and restitution of multiple tissue lineages (224–226). MSCs regenerative properties are mediated by their ability to infiltrate and engraft injured areas, where they reduce inflammation, promote angiogenesis, prevent apoptosis, improve scar formation, and mediate tissue remodeling via the paracrine secretion of chemokines and growth factors (227, 228). These pluripotent stem cells are capable of reprogramming into differentiated phenotypes participating in regenerative and reparative programs of most tissues and organs (229–234).

Within the realm of impaired diabetic wound healing, MSCs allogenic transplantation has demonstrated significant healing improvement at both experimental (235–237), and clinical scenarios (238–240). It is controversial however whether autologous MSCs regenerative and pro-survival capabilities, are conserved within diabetic organisms given the complex, invasive, and “metastazing” pathophysiology of this disease (241, 242). Diabetic animals show a lower number of circulating MSCs with deteriorated proliferation and survival capabilities. Furthermore, these cells also exhibit an impaired recruitment and insufficient engraftment response within diabetic wounds (241, 243). Similarly, diabetic-associated MSCs dysfunctionalities are described in humans. Diabetic individuals show lower levels of CD34–/CD133+/KDR+ endothelial progenitor cells (EPC) counts along with higher apoptotic rates of circulating EPC (244). Ultimately, MSCs population is affected at every physiological respect by the cytotoxic diabetic environment (245, 246), which turns it prone to replicative senescence and limits its multipotency expansion ability (69, 247, 248).

**Figure 4:**
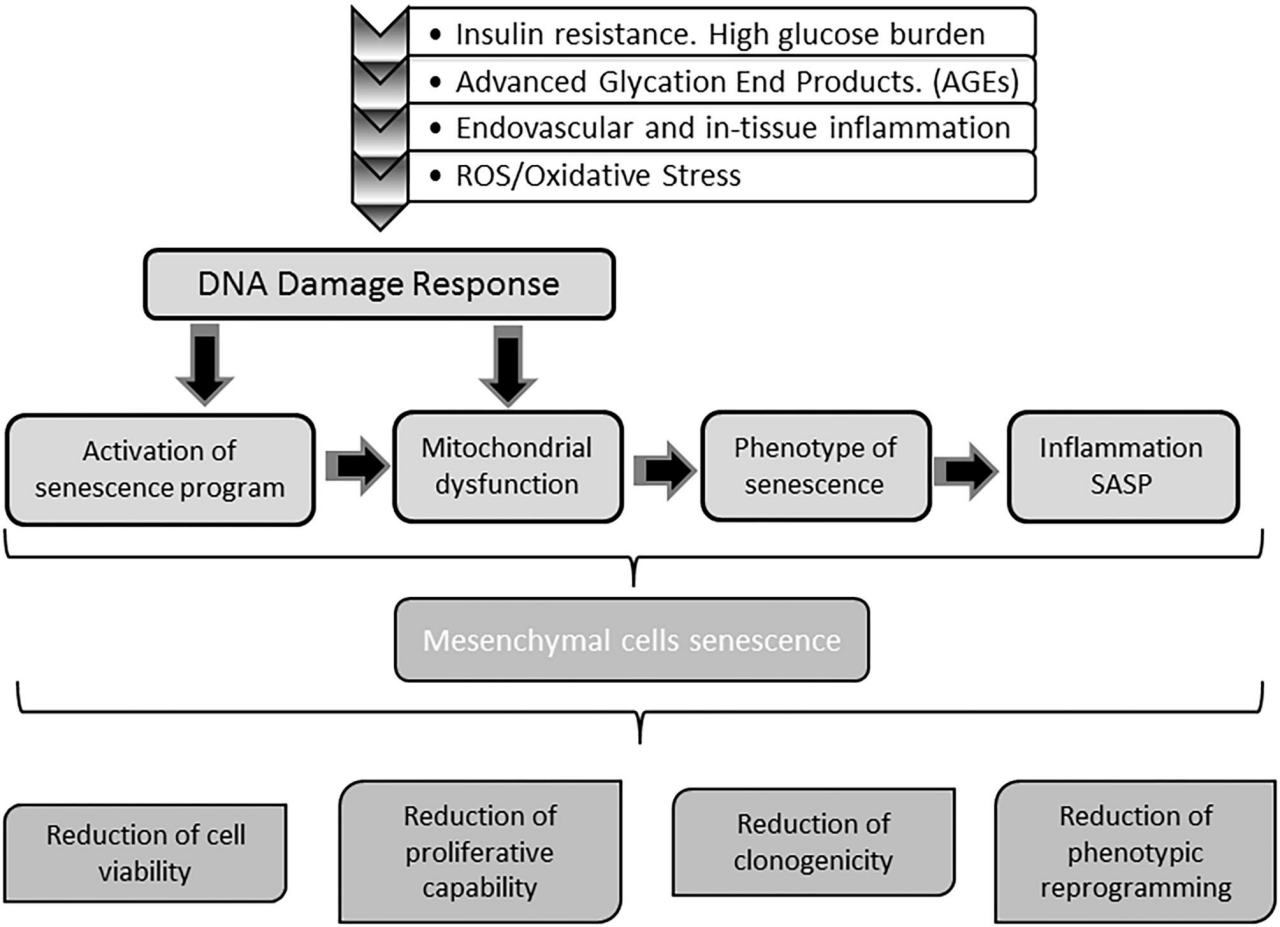
Impact of diabetes on stem cells physiology. Mesenchymal stem cells (MSC) are also targeted by the molecular gluco-oxidative hostile environment of diabetes. The mechanisms and pathways whereby MSC become senescent are the same senescent stressors buffeting differentiated somatic cells. Senescence of MSC impairs stem cells physiology and indirectly that of somatic cells in both mitotic and post-mitotic populations. Upon senescence, stem cells circulating pool is reduced and their ability to engraft injured tissues is also compromised. Concurrently, the capabilities of these cells to assist in wound repair as to participate in epithelial cells populations turnover is also impaired. Globally speaking, with diabetes MSC senescence the tissue's resilience based on the self-renewal potential are undermined.

Converging evidences conclusively demonstrate how toxic high glucose burden may be for survival, differentiation plasticity, and regenerative competence for different stem cells lineages (249–253). MSCs are markedly susceptible to hyperglycemia-induced ROS/oxidative stress-mediated senescence (245, 254). The perpetuative vicious circle integrated by hyperglycemia/mitochondrial dysfunction/oxidative stress appears again as a master driver to MSCs senescence (255, 256). In this scenario, ROS/oxidative stress activate a p53 program with the ensued cells proliferative arrest as described for mitotic differentiated stem cells (257). Thus, as for other cells stirpes, redox imbalance is a key factor in imposing a premature senescence program (249, 256). Accordingly, the diabetic pro-oxidative environment is a major contributing factor for premature MSCs senescence and functional demise (246). Ultimately, MSCs are victims like other somatic cells of the hyperglycemia-determined oxidative stress with the ensued mitochondrial and genomic DNA damage, the onset of a pathogenic SASP, and the paracrine inflammatory reaction (256, 258, 259). The continuous surge of cytotoxic and pro-inflammatory mediators of the diabetic environment, render MSCs numerical and functionally deficitary for the repair process of diabetic ulcers (260). As summarized in Figure 4, MSCs do not escape from the entangled and interconnected mechanisms that drive cellular senescence in diabetes.

## Concluding Remarks

Diabetes is a pre-conditioning and powerful driver of organismal aging. The biological foundations of aging primarily involve cellular senescence and diabetes is plethoric in senescence-driving factors. Definitively, high glucose concentrations are the proximal trigger for all the subsequent and long lasting molecular by-products, which turn diabetes an archetypal disease of “aging hallmarks.”

Cellular senescence is considered today as intimately related to diabetes complications progression, which are a major cause for poor health span and decreased lifespan. Cellular senescence is also a major steering factor for diabetic poor wound healing response. Pathogenically speaking, fibroblasts, endothelial cells, and keratinocytes are targeted by a broad range of diabetes-related pro-senescence factors. Most interestingly, these cells become and remain stubbornly refractory to migrate, proliferate, and secrete matrix ingredients both *in vivo* and *in vitro*—For the later, suggesting the existence of a sculptured epigenomic memory that along with the wound local senescence secretome creates the long-lasting senescent cells society within the wound. This society with its active pro-aging secretome contributes to wound chronicity perpetuation. Diabetic wounds chronicity phenotype is also impinged by two major integrated and cooperative forces: “metabolic memory” and “senescence memory” —both endowed with the ability to manipulate cells behavior. A frightened stamp of diabetic foot wounds is their recurrences short time after re-epithelialization. We deem that the episode of this *in situ* re-ulceration is an illustrative reflection of the consequence of a long-subsisting senescence society with imprinted “memories” and an active diabetic SASP secretome.

Both bone marrow and tissue niches of MSCs become prematurely senescent under the pressure of the diabetes biochemical environment. These cells are extremely sensitive to glycoxidation products as to oxidative stress, which reduces the population of biological competent cells for epithelial organs self-renewal, for tissue maintenance, and ultimately to actively participate in tissue injury repair, especially in post-mitotic organs.

All together these molecular and cellular senescence traits underlie the diabetic's organismal illnesses and tissues vulnerabilities. Although this has been a long-sought goal we deem that diabetes still waits for a pharmacological re-interpretation. It is likely that establishing “lines of treatments” beyond glucose-lowering medications and improving insulin sensitivity, will translate in great therapeutic value for reducing, and even preventing diabetic complications including the torpid healing phenotype. These “lines of treatments” may represent a cluster of tactics addressed to control cellular senescence and ultimately organismal aging. Alluring targets could be:

1. The molecular basis of diabetic metabolic and senescence memories and their epigenetic signatures for selective pharmacological manipulation.
2. Pharmacological compounds with the ability to selectively modify the histone landscape in a complication and tissue-specific manner.
3. Mitochondria are a sensitive, appealing, and unexplored therapeutic target field. Pharmacological tools aimed to preserve the axis telomeres-mitochondrial function and accordingly to regulate its biogenesis could be promising. “Mitochondrio-therapy” could not only revolutionize diabetes, but expand to cancer and neurodegenerative diseases.
4. Finally, the identification of therapeutic interventions acting as selective senolytics is justified.

Harnessing and tempering diabetic cellular senescence in an appropriate therapeutic opportunity window, will be of paramount clinical and societal significance.

## Author Contributions

JB-A, GG-N, and PV-S contributed equally to the initial project conception. JB-A wrote the initial draft of the manuscript. DG, YM-M, NR-R, IM-J, SH-G, and JB-S contributed to analysis and interpretation of all the references and data related to this MS. MB-V was in charge of the preparation of the manuscript and the whole editorial process. JB-A and JB-S contributed equally to data collection. All authors contributed to the interpretation, critical review, and approved the final version of the manuscript.

## Conflict of Interest

The authors declare that the research was conducted in the absence of any commercial or financial relationships that could be construed as a potential conflict of interest.
